# Endolithic fungal diversity is present in the unique phosphatized rocks of an environmentally extreme equatorial archipelago revealed by DNA amplicon metagenomics

**DOI:** 10.1007/s42770-025-01840-5

**Published:** 2026-01-28

**Authors:** Laucélly Bárbara Avelar Rocha, Vívian Nicolau Gonçalves, Fábio Soares de Oliveira, Guilherme Resende Corrêa, Eduardo Osório Senra, Eduardo Baudson Duarte, Fabyano A. C. Lopes, Micheline C. Silva, Peter Convey, Paulo E. A. S. Câmara, Luiz Henrique Rosa

**Affiliations:** 1 Departamento de Microbiologia, Universidade Federal de Minas Gerais, Belo Horizonte, Minas Gerais Brazil; 2 Departamento de Geografia, Universidade Federal de Minas Gerais, Belo Horizonte, Brazil; 3 Universidade Federal de Uberlândia, Uberlândia, Minas Gerais Brazil; 4 Instituto Federal do Espírito Santo, Campus Nova Venécia, Vitória, Brazil; 5 Laboratório de Microbiologia, Universidade Federal do Tocantins, Porto Nacional, Brazil; 6 Departamento de Botânica, Universidade de Brasília, Brasília, Brazil; 7 British Antarctic Survey, NERC, High Cross, Madingley Road, Cambridge, CB3 0ET UK; 8 Department of Zoology, University of Johannesburg, Auckland Park 2006, Johannesburg, South Africa; 9 School of Biosciences, University of Birmingham, Edgbaston, Birmingham, B15 2TT UK; 10 Laboratório de Microbiologia Polar e Conexões Tropicais, Departamento de Microbiologia, Instituto de Ciências Biológicas, Universidade Federal de Minas Gerais, P. O. Box 486, Belo Horizonte, CEP 31270-901 MG Brazil

**Keywords:** Atlantic ocean, Environmental DNA, Extremophiles, Rocks, Taxonomy

## Abstract

We evaluated endolithic fungal diversity associated with rocks sampled at the polyextreme Brazilian São Pedro and São Paulo archipelago using a DNA amplicon metagenomics approach. We detected 808,547 fungal DNA reads grouped into 92 amplicon sequence variants (ASVs). The rocks sampled were geologically characterized as mylonitized peridotites, serpentinized peridotites, and carbonate-matrix sedimentary breccias. *Ascomycota* was the dominant phylum, followed by *Basidiomycota*, *Mucoromycota*, *Mortierellomycota* and *Chytridiomycota*. *Hortaea werneckii*, *Cladosporium* sp., *Simplicillium* sp., *Blastobotrys serpentis*, *Penicillium* sp., *P. simplicissimum*, *Malassezia restricta*, *Ascomycota* sp., *Verrucariaceae* sp., and Fungal sp. were the dominant assigned taxa. The endolithic assemblages displayed moderate to low diversity indices. Among the fungal community, only the dominant Fungal sp. occurred in all samples. The data obtained in our environmental DNA (eDNA) amplicon metagenomics approach suggest that the rocks of the isolated equatorial São Pedro and São Paulo archipelago host a complex fungal diversity, including taxa regarded to be cosmopolitan, extremophilic hypersaline and xerophilic, plant pathogens, and human/animal opportunistic pathogens. As eDNA studies do not confirm the presence of viable organisms or propagules, further research using culturing approaches is now required to develop strategies to recover these fungi for physiological, biogeochemical, genetic and potential biotechnological studies.

## Introduction

The Brazilian archipelago of São Pedro and São Paulo is characterized by the high temperatures typical of the equatorial regions. The archipelago consists of several small and sparsely vegetated rocky islands that face the impact of powerful waves, intense solar radiation, low availability of fresh water, and high salinity [1]. Due to the extreme conditions typifying the archipelago, the limited diversity of terrestrial organisms present must withstand polyextremophilic conditions. Microorganisms are a particularly notable element of this diversity.

The São Pedro and São Paulo archipelago is geologically unique among Atlantic oceanic archipelagos, as it is uplifted ocean floor consisting of mylonitized and serpentinized peridotites along with carbonate sedimentary rocks [2–6]. The archipelago also contains Quaternary sedimentary rocks of the São Pedro and São Paulo Formation [5, 6]. These include moderately to poorly sorted conglomerates (Atobás Unit) and sandstones (Viuvinhas Unit), composed of subrounded to subangular ultramafic lithoclasts and calcareous bioclasts, cemented by carbonate [6]. Unlike most mid-Atlantic islands and archipelagos, which originate from volcanic activity, the São Pedro and São Paulo Archipelago is composed of ultramafic infracrustal rocks derived from the Earth’s mantle, making it a geological anomaly on a global scale. This exceptional setting offers rare insight into deep-seated tectonic and geochemical processes, reinforcing the scientific and geological importance of the archipelago.

Peridotitic rocks in the archipelago occur in two distinct types, differing in texture, structure and color. Mylonitized peridotites, primarily found in northwestern Belmonte Island, are massive, aphanitic and highly resistant, ranging in color from grayish-white to greenish-gray. In contrast, serpentinized peridotites are highly fractured, heterogeneous and softer, blending, colors from grayish-white and greenish-gray to reddish-brown [5]. Tectonic activity following mylonitization led to extensive rock fragmentation, allowing fluid infiltration, which triggered serpentinization and induced microchemical and structural transformations in the peridotites [7].

Among extremophiles, microbes of the Kingdom Fungi include many taxa that are exceptionally well adapted for survival in diverse harsh environments globally [8]. They are capable of tolerating stresses such as extreme temperatures, prolonged desiccation, and intense solar radiation by utilizing features of their physiology and morphology, including the production of various specialized secondary metabolites [9]. Among extremophilic fungi, rock-inhabiting fungi (RIF), known as endolithic fungi, represent a generally poorly studied group that has gained attention due to their ability to survive under polyextreme conditions in some of the harshest environments on the planet [8, 10]. The São Pedro and São Paulo Archipelago, a polyextreme equatorial Brazilian region, displays unique geology. As the resident fungal (RF) community of these rocks is currently unknown, this study documented the fungal diversity present using DNA amplicon metagenomics through high-throughput sequencing (HTS).

## Materials and methods

### Rock sampling and processing

**Fig. 1:**
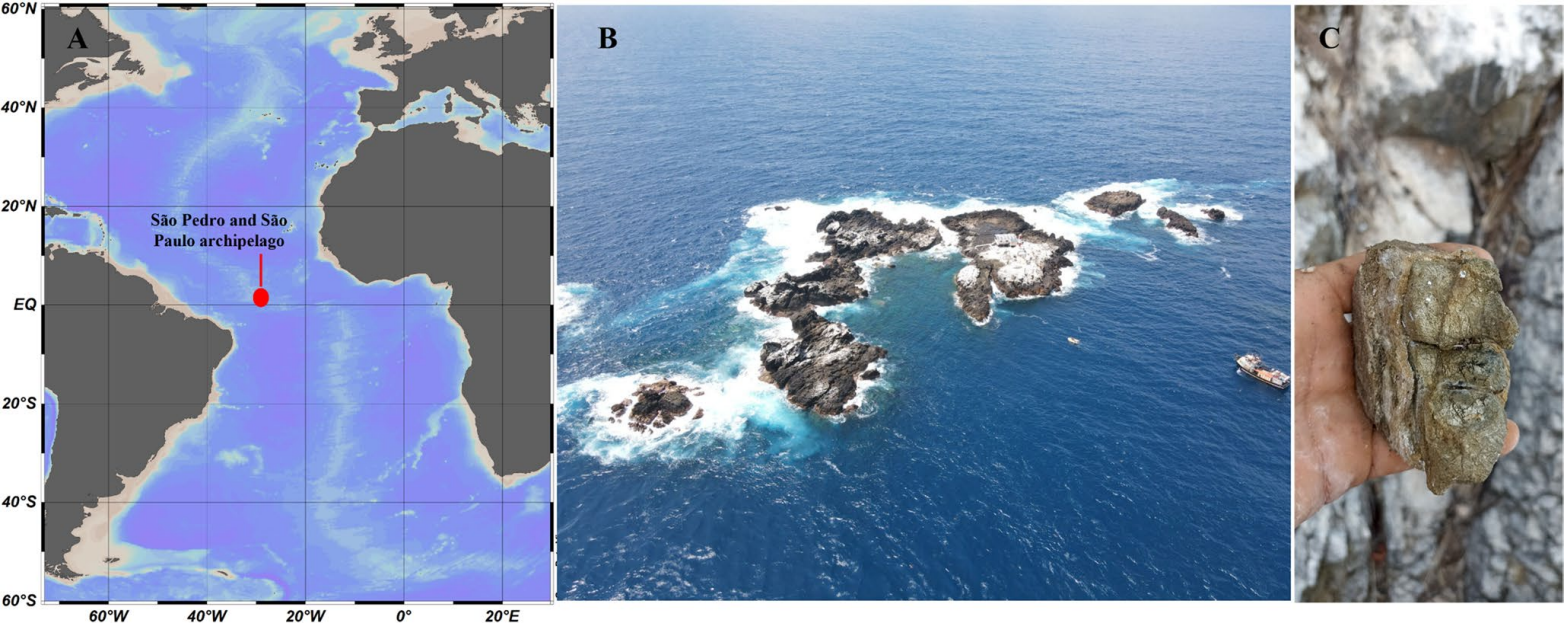
(**A**) Location (red dot) of the São Pedro and São Paulo Archipelago (00°55′1″N; 29°20′45″W) in the equatorial Atlantic Ocean, (**B**) aerial photograph of the entire archipelago, and (**C**) example of rock sampled in the archipelago. Photographs **A** and **C** by E.O. Senra

Samples were obtained in August and September 2022 at six sites on the São Pedro and São Paulo archipelago (0°54’59” S; 29°20’44” W), located in the equatorial Atlantic Ocean about 1,000 km from the Brazilian mainland (Fig. 1). All three known rock types were sampled: mylonitized peridotites (samples 1 and 2), serpentinized peridotites (samples 3, 4 and 5), and carbonate-matrix sedimentary breccias (sample 6). Samples were collected using sterile gloves, sealed in Whirl-Pak bags (Nasco, Ft. Atkinson, WI, USA) and kept at -20 °C immediately after sampling and during transportation to the laboratory at the Federal University of Minas Gerais, Brazil, where the samples were processed.

The rocks were fragmented and pulverized using a Bosch GSB13 RE (Brazil) drill with 4–5 mm drill bits. The rocks were surface-sterilized, and drill bits sterilized between processing different samples, by spraying with absolute ethanol and flame-sterilization. To minimize the risk of contamination, this process was carried out in a laminar flow cabinet. The pulverized rock samples (500 mg) were packaged in sterilized 2 mL tubes in triplicate for DNA extraction. The samples were stored at -20 °C in the laboratory of the Federal University of Minas Gerais, Brazil, until total DNA extraction.

### Geological analysis

Petrographic analyses were completed to confirm the types of rocks and their main features. Sample descriptions were made at macro- and microscopic scales, using “hand samples” and “thin sections”, respectively. The mineral constituents, colors, textures and structures were analyzed. In the thin sections, optical microscopic investigations were carried out using a Zeiss trinocular optical microscope (Axiophot model) with an integrated digital camera. Additionally, total geochemical composition analyses were performed using acid digestion of powdered samples. Aliquots of 0.100 g were digested in closed Teflon containers with 5 mL ultrapure HNO₃ (67–69%) and 2 mL ultrapure HF (47–51%) using a microwave system (Ethos One, Milestone). The digestion process involved heating to 200 °C for 120 min. The resulting solutions were transferred to polypropylene tubes, diluted to 50 mL with Milli-Q water, and further 10-fold diluted before analysis. A total of 40 elements were quantified, including major, minor, trace and rare earth elements. Blank samples and certified reference materials (IAEA-158, MESS-2, and TORT-2) were used to ensure accuracy and quality control through ICP-OES/MS analysis. Due to the limited amount of collected material, the samples were grouped according to the three rock types: mylonitized peridotites (samples 1 and 2), serpentinized peridotites (samples 3, 4 and 5), and carbonate-matrix sedimentary breccias (sample 6).

### DNA extraction, illumina library construction and sequencing

Three replicate sub-samples (approximately 500 µg) of each of samples 1, 2, 3, 4, 5 and 6 were used for DNA extraction. Total DNA was extracted from these using the FastDNA Spin Kit for Soil (MPBIO, Ohio, USA) under strict contamination control conditions, following the manufacturer’s instructions. DNA quality was analyzed using agarose gel electrophoresis (1% agarose in 1 × Trisborate-EDTA) and then quantified using the Quanti- iT™ Pico Green dsDNA Assay (Invitrogen). The extracted DNA was used as template for generating PCR amplicons. The internal transcribed spacer 2 (ITS2) of the nuclear ribosomal DNA was used as a DNA barcode for molecular species identification [11–13]. PCR amplicons generated using the universal primers ITS3 and ITS4 [14] were sequenced commercially by Macrogen Inc. (South Korea) using high-throughput paired-end sequencing (2 × 300 bp) on a MiSeq System (Illumina), using the MiSeq Reagent Kit v3 (600 cycles).

### Data analysis and fungal identification

DNA quality analysis was carried out using BBDuk v. 38.87 in BBmap software [15] with the following parameters: Illumina adapters removing (Illumina artefacts and the PhiX Control v3 Library); ktrim = l; k = 23; mink = 11; hdist = 1; minlen = 50; tpe; tbo; qtrim = rl; trimq = 20; ftm = 5; maq = 20. The remaining sequences were imported to QIIME2 version 2025.5 (https://qiime2.org/) for bioinformatics analyses [16]. The qiime2-dada2 plugin was used for filtering, dereplication, turning paired-end fastq files into merged and removal of chimeras, using default parameters [17]. Taxonomic assignments were determined for amplicon sequence variants (ASVs) in three steps. First, ASVs were classified using the qiime2-feature-classifier [18] classify-sklearn against the UNITE Eukaryotes ITS database version 10.0 [19]. Second, remaining unclassified ASVs were filtered and aligned against the filtered NCBI non-redundant nucleotide sequences (nt) database (October 2021) using BLASTn [20] with default parameters; the nt database was filtered using the following keywords: “ITS1”, “ITS2”, “Internal transcribed spacer” and “internal transcribed spacer”. Third, output files from BLASTn [21] were imported to MEGAN6 [22] and taxonomic assignments were performed using the "megan-nucl-Feb2022.db” mapping file with default parameters and trained with Naive Bayes classifier and a confidence threshold of 98.5%. Taxonomic profiles were plotted using the Krona [19]. Sequences have been submitted to GenBank under the accession numbers SAMN37305790-SAMN37305802.

Many factors, including extraction, PCR and primer bias, can affect the number of reads obtained [22], and thus lead to misinterpretation of absolute abundances [23]. However, Giner et al. [24] concluded that such biases did not affect the proportionality between reads and cell abundance, implying that more reads are linked with higher abundance [25, 26]. Therefore, for comparative purposes, we used the number of reads as a proxy for relative abundance. Fungal classification followed Kirk [27], Tedersoo et al. [28], MycoBank (http://www.mycobank.org) and the Index Fungorum (http://www.indexfungorum.org).

### Fungal diversity

The number of DNA reads and relative abundances of the ASVs were used to quantify the fungal taxa present in the samples. Fungal ASVs with relative abundance >1% were considered dominant and those < 1% as minor (rare) components of the fungal community [13]. The relative abundances were used to quantify taxon diversity, richness, and dominance, using the following indices: (i) Fisher’s α, (ii) Margalef’s and (iii) Simpson’s, respectively. Species accumulation curves were obtained using the Mao Tao index. All results were obtained with 95% confidence, and bootstrap values were calculated from 1,000 replicates using the PAST computer program 1.90 [29]. Venn diagrams were prepared following Bardou et al. [30] to visualise the fungal assemblages present in the different sampling sites.

## Results

### Geological rock analysis

**Fig. 2:**
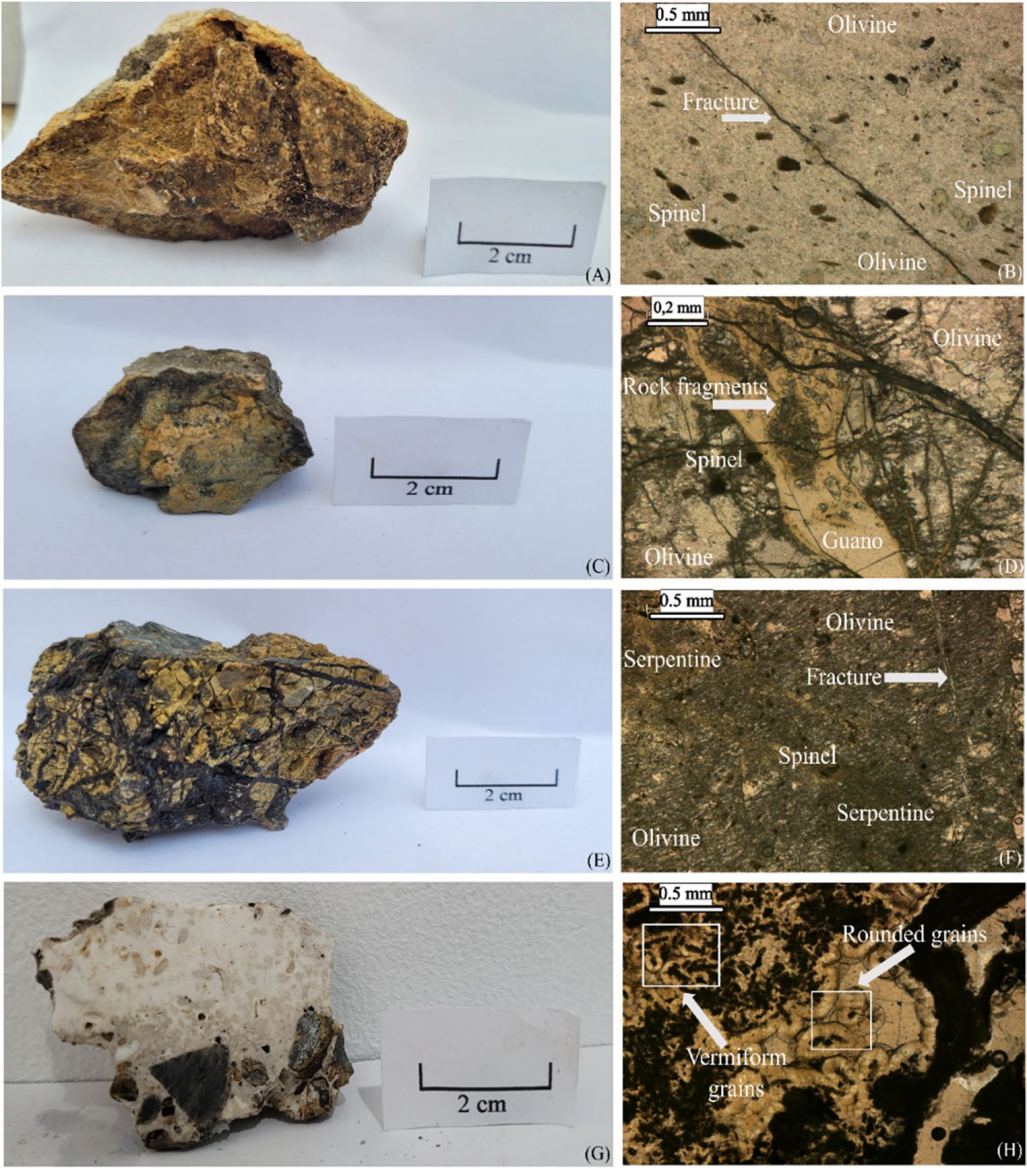
Geological features of rocks sampled in the São Pedro and São Paulo Archipelago. (**A**) and (**B**) represent rock sample 1, (**C**) and (**D**) sample 2, (**E**) and (**F**) Sample 5, and (**G**) and (**H**) sample 6

The mylonitized peridotites (samples 1 and 2) exhibited minimal fracturing and displayed a heterogeneous orange coloration interspersed with brownish, greenish and whitish portions (Fig. 2A and C). Microscopically, they consist of a dominant fine-grained olivine matrix surrounding larger olivine grains and brown spinel, forming an equigranular porphyroclastic texture (Fig. 2B and D). Petrographic observations indicate that these rocks have been affected by bird guano deposition, as the guano infiltrates fractures in the peridotite, leading to phosphate mineralization. This process results in light yellow to cream-colored phosphate phases observed in the samples (Fig. 2D). The serpentinized peridotite basement exhibited a diverse color range, including yellow, black, brown and gray, with an extensive fracture network (Fig. 2E). The rocks displayed an equigranular porphyroclastic texture, characterized by fine- to medium-grained olivine and fibrous serpentine (Fig. 2F), along with minor amounts of fine brown spinel grains.

Macroscopically, the sedimentary breccia presented a sandy-silty texture, incorporating small peridotite lithoclasts and biological components. It had a whitish coloration with brown, orange, and black portions (Fig. 2G). It exhibited considerably less fracturing than observed in mylonitized and serpentinized peridotites. Under optical microscopy, the grains exhibited a spherical to ellipsoidal structure, resembling ooids (Fig. 2H), and included small peridotite lithoclasts and biological components. Millimeter-thick fractures were irregularly filled with vermiform-shaped crystals. The fracture infill and the coatings of rounded spherulitic crystals exhibited variations in color, texture, and structure, suggesting potential mineralogical and/or chemical differences.

Geochemical analyses (Table 1) showed that the mylonitised peridotites (samples 1 and 2), the serpentinized peridotite (sample 3, 4 and 5) and the sedimentary rock (sample 6) were mainly composed of SiO_2_, ranging from 33.86% in the sedimentary breccia to 50.78% in the mylonitised peridotite. MgO content also made a significant contibution, ranging from 22.7% to 34.49%, associated with mafic silicate minerals. Content of CaO and LOI were highest in the sedimentary breccia, 10.93% and 12.18%, respectively, mainly attributed to the presence of carbonates and the decarbonation of adsorbed H_2_O + bound H_2_O + CO_2_. Conversely, substantial variation in the CaO and LOI contents was apparent between the mylonitised peridotites and the serpentinised peridotite, ranging from 1.66% to 7.81% and 1.68% to 9.42%, respectively. This variation is presumably associated with the presence of serpentines.

**Table 1:** Physicochemical analysis and diversity indices of fungal assemblages detected from each of the rock samples obtained in the São Pedro and São Paulo Archipelago

	Rock samples					
	Mylonitized peridotites		Serpentinized peridotites			Carbonate-matrix sedimentary breccias
**Physicochemical analysis**	**1**	**2**	**3**	**4**	**5**	**6**
SiO_2_ (%)	45.43		46.35			33.86
Al_2_O_3_ (%)	1.74		1.89			1.46
CaO (%)	4.735		2.29			10.93
Na_2_O (%)	0.48		0.25			0.78
K_2_O (%)	0.265		0.08			0.22
MnO (%)	0.1		0.14			0.22
MgO (%)	28.595		32.22			24.59
TiO_2_ (%)	0.045		0.05			0.02
Fe_2_O_3_ (%)	8.565		7.05			5.77
P_2_O_5_ (%)	6.25		0.25			9.98
LOI (%)	3.79		9.42			12.18
As (ppm)	< LQ		< LQ			< LQ
Ba (ppm)	19.15		6.93			18.2
Bi (ppm)	< LQ		< LQ			< LQ
Cd (ppm)	2.02		< LQ			< LQ
Co (ppm)	80.695		91.49			205
Cr (ppm)	2759		2336			1935
Cu (ppm)	9.93		< LQ			179
Li (ppm)	< LQ		< LQ			< LQ
Mo (ppm)	2.84		< LQ			< LQ
Ni (ppm)	1476.5		1844			1488
Sc (ppm)	9.275		9.71			5.82
Sr (ppm)	260.27		22.27			928
Th (ppm)	9.92		< LQ			12.72
V (ppm)	45.845		48.97			31.01
Y (ppm)	< LQ		< LQ			0.499
Zn (ppm)	149.275		69.3			217
Be (ppm)	< LQ		< LQ			< LQ
Sb (ppm)	< LQ		< LQ			< LQ
Zr (ppm)	3.96		3.61			2.9
Pb (ppm)	< LQ		< LQ			< LQ
S (ppm)	1792.5		1046			6779
**Diversity indices**	**1**	**2**	**3**	**4**	**5**	**6**
Number of fungal ASVs	15	36	12	8	50	14
Number of assigned ASVs	92,036	162,115	75,777	183,670	257,934	37,015
Fisher’s α	1.37	3.33	1.07	0.64	4.57	1.37
Margalef	1.22	2.92	0.98	0.57	3.93	1.23
Simpson	0.73	0.37	0.33	0.03	0.32	0.61

< *LQ* below the limit of quantification, *LOI* loss on ignition, *ASVs* amplicon sequence variants

The Fe_2_O_3_ content was lowest in the sedimentary breccia (5.77%), with the serpentinized peridotite (7.05%) exhibiting the next highest content and the highest content in the mylonitised peridotites (8.45–8.68%). The sedimentary breccia and one sample of mylonitized peridotite had the highest levels of P_2_O_5_, 9.98% and 11.64%, respectively. Levels did not exceed 1% in the other rocks. All rock types had low total Al_2_O_3_ contents, ranging from 1.46% to 1.89%, while the total contents of Na_2_O, K_2_O, MnO and Ti_2_O were < 1% in all rocks analysed.

The maximum Cr contents (2336 to 2891 ppm) were found in the ultramafic rocks (mylonitized peridotite and serpentinized peridotite), followed by the sedimentary breccia with < 2000 ppm (Table 1). The highest Co, Cu and S contents were associated with the sedimentary breccia (205, 179 and 6779 ppm, respectively). The highest Ni contents (> 1800 ppm) were present in mylonitised peridotite and serpentinized peridotite, while the sedimentary breccia had the highest Sr and Zn contents (928 ppm and 217 ppm) and the samples of mylonitised peridotite (506 ppm and 233 ppm). Total V content exhibited limited variation across the samples (31.01 to 49.79 ppm), although with lower concentrations in the sedimentary breccia. Conversely, Ba content demonstrated considerable variability (5.48 to 32.82 ppm), with the highest concentrations found in one sample of mylonitised peridotite and in the sedimentary breccia. All samples exhibited low concentrations of Sc, Th and Zr, while the concentrations of As, Bi, Cd, Li, Mo, Y, Be, Sb and Pb were below the limit of quantification (< LQ) in most samples.

### Fungal identification

A total of 808,547 fungal DNA reads were obtained, which were assigned to 92 amplicon sequence variants (ASVs) (Table 2). *Ascomycota* was the dominant phylum, followed by *Basidiomycota*, *Mucoromycota*, *Mortierellomycota*, and *Chytridiomycota*, in rank order. *Hortaea werneckii*, *Cladosporium* sp., *Simplicillium* sp., *Blastobotrys serpentis*, *Penicillium* sp., *Penicillium simplicissimum*, *Malassezia restricta*, *Ascomycota* sp., *Verrucariaceae* sp., and Fungal sp. were the taxa classified as dominant (relative abundance ≥ 1%). A further 84 fungal ASVs formed minor components of the assigned fungal community. 

**Table 2:**
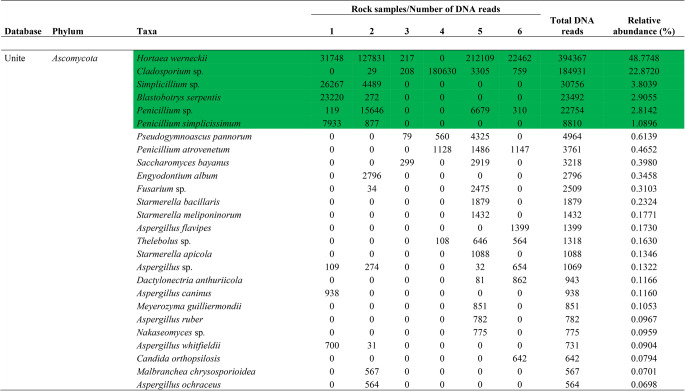

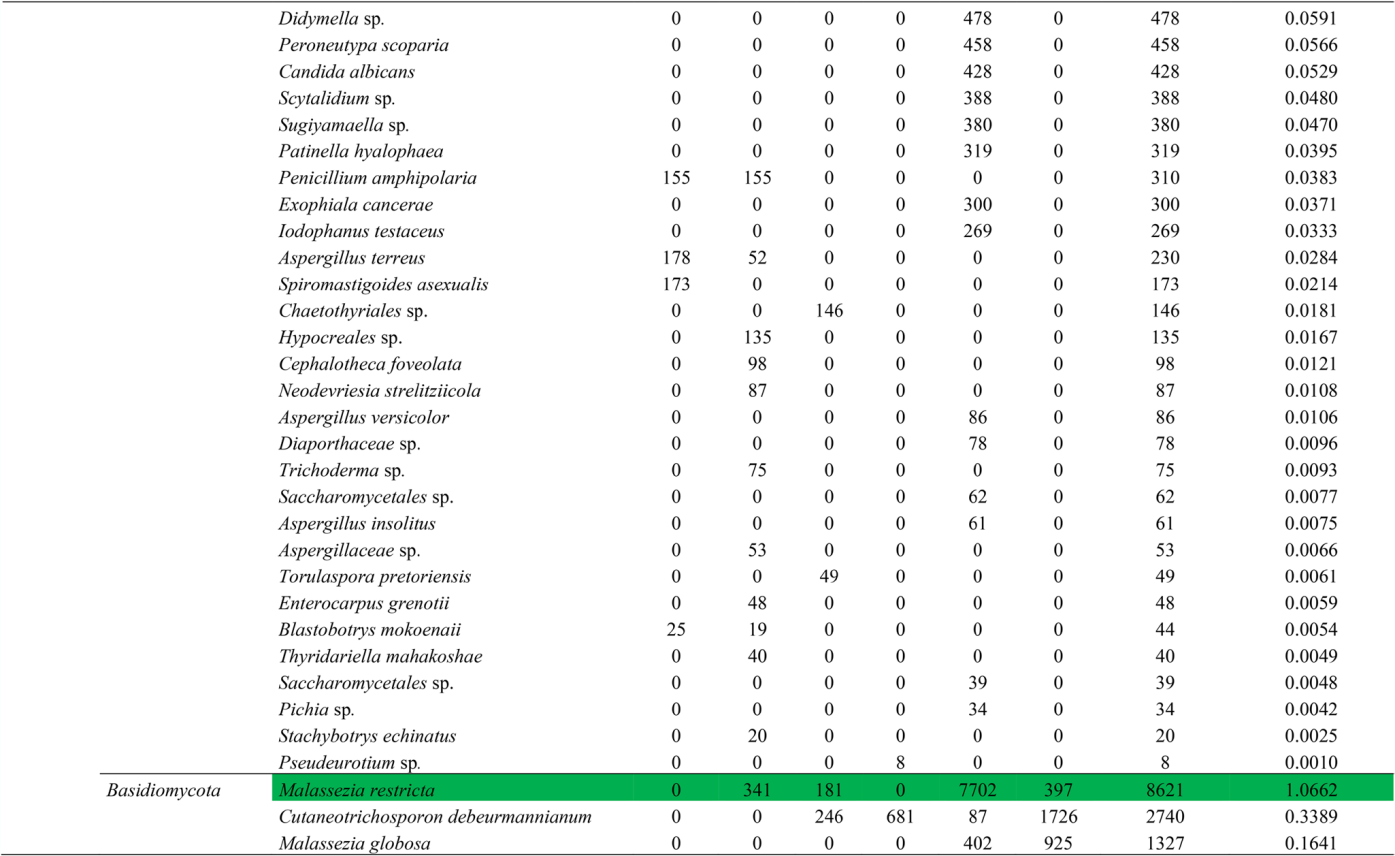

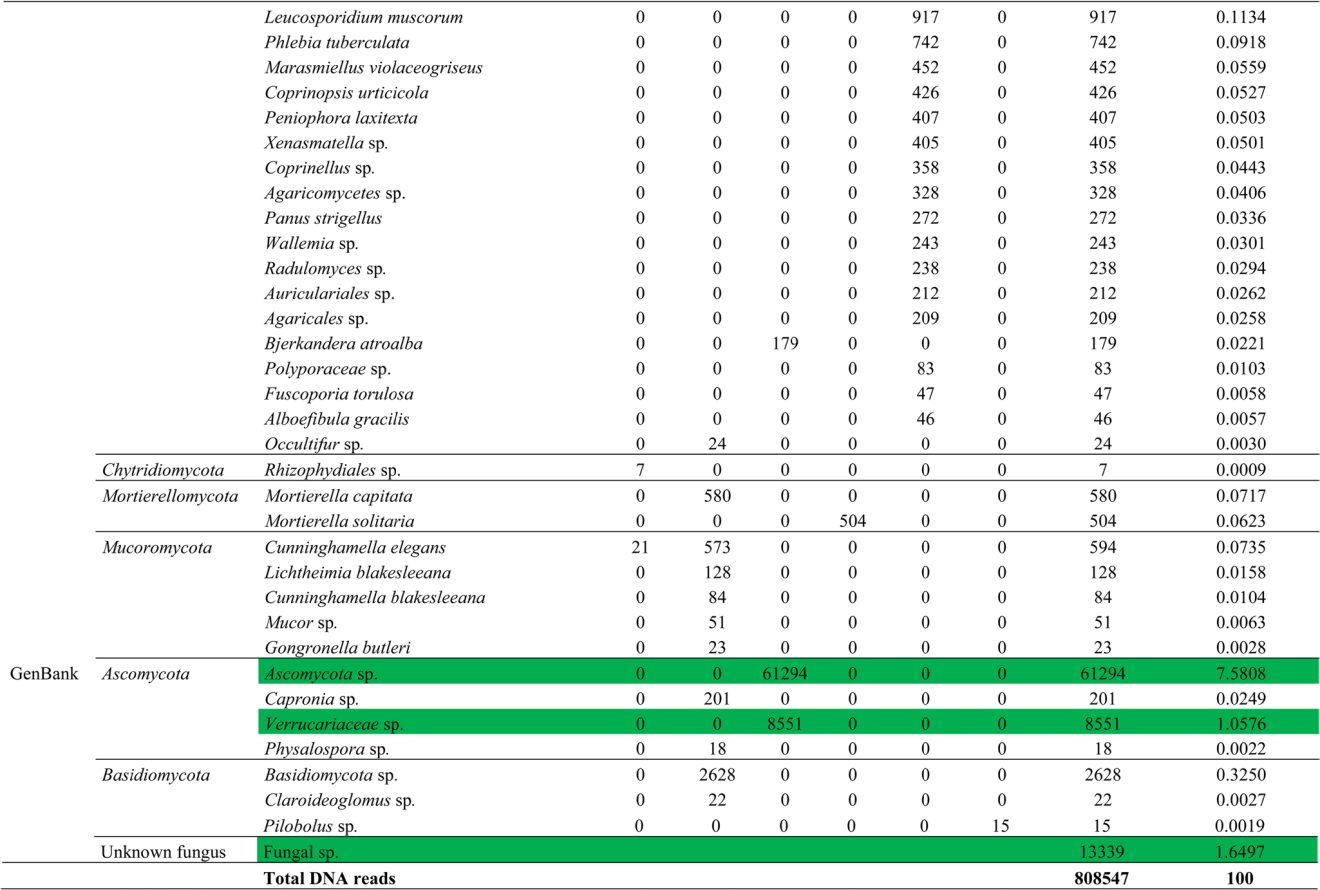
Abundances of endolithic fungal amplicon sequence variants (ASVs) detected in rock samples from the São Pedro and São Paulo archipelago

Dominant taxa with relative abundance >1% are shown in green

### Fungal diversity and distribution

**Fig. 3:**
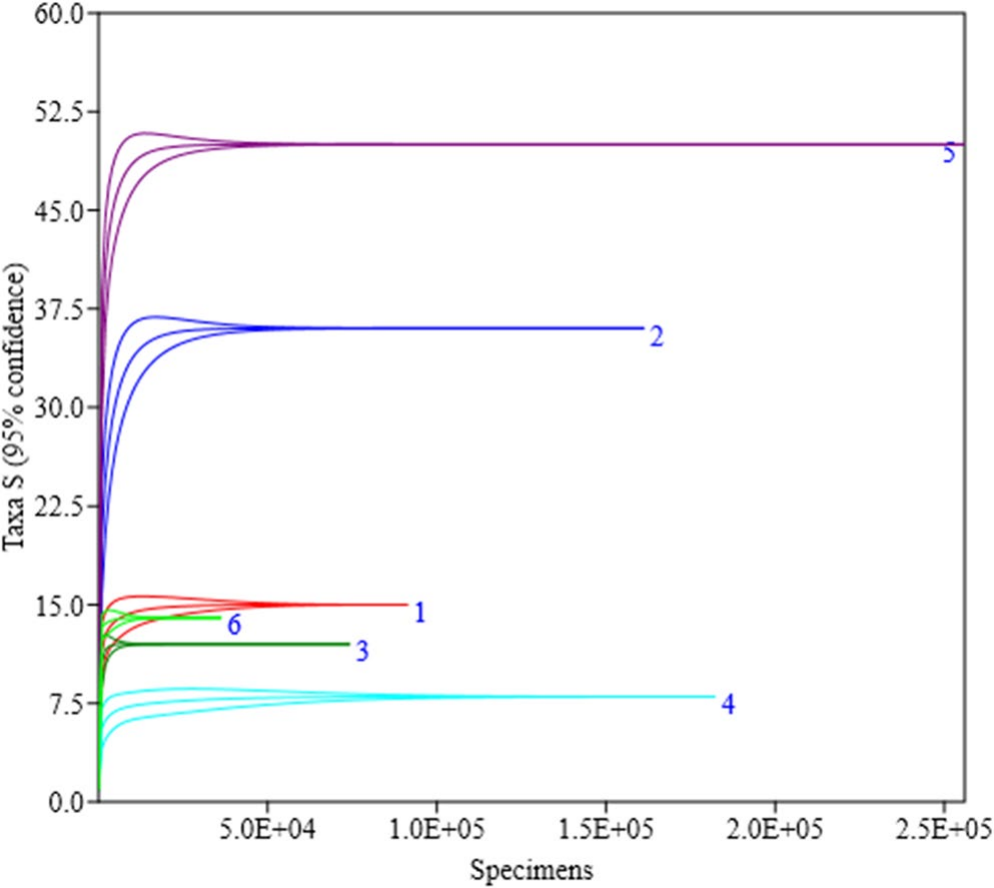
Rarefaction curves (Mao Tao index) for the fungal assemblages detected from each of the six rock samples obtained from the São Pedro and São Paulo Archipelago

**Fig. 4:**
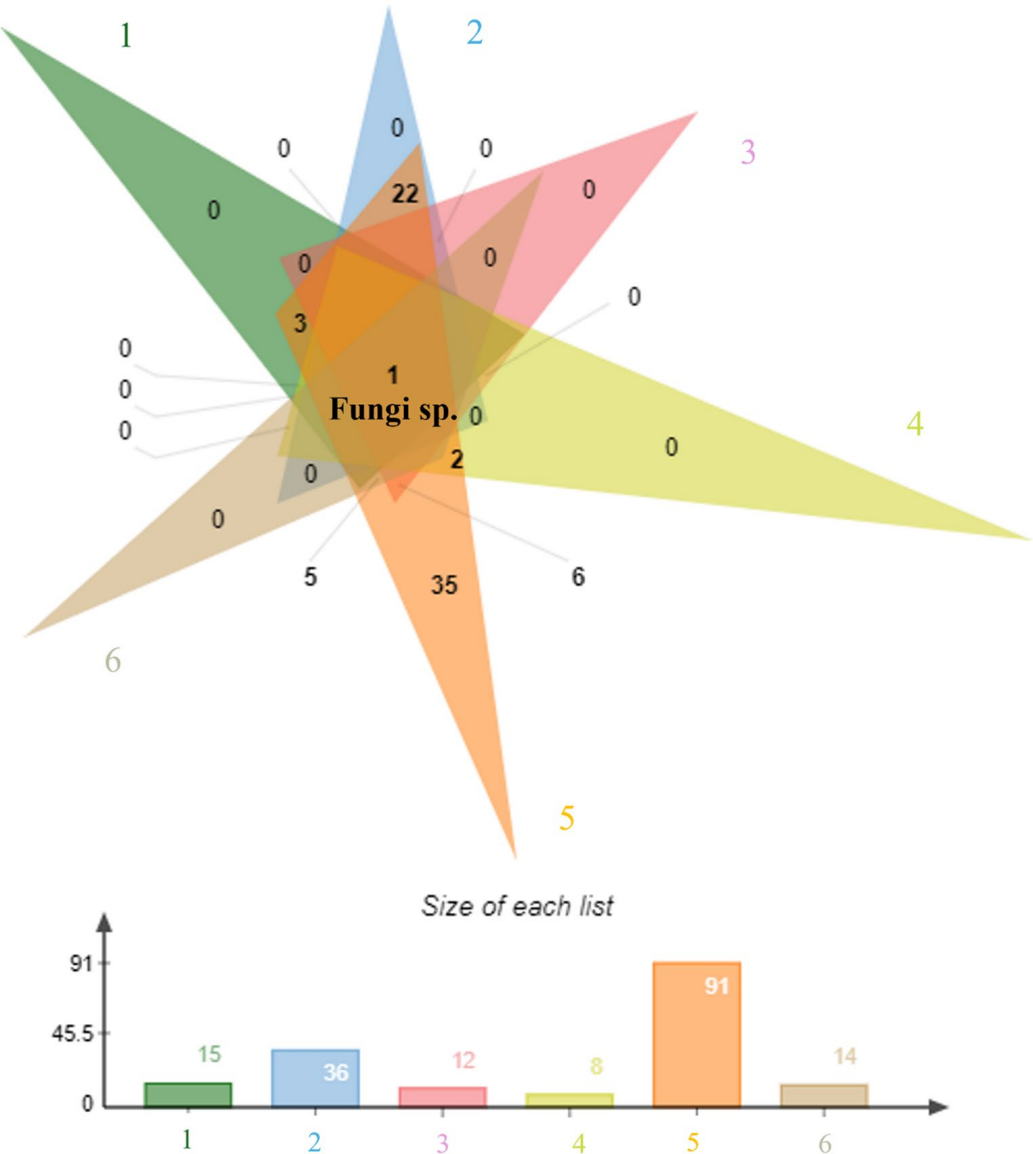
Venn diagram illustrating assigned fungal ASV diversities across the six rock samples obtained from the São Pedro and São Paulo Archipelago

The Mao Tao rarefaction curves reached asymptote for all fungal assemblages from the six individual sampled rocks, indicating that the majority of the diversity present was detected (Fig. 3). The rock assemblages displayed moderate to low diversity indices, although varying between the samples (Table 1). The highest number of ASVs, diversity (Fisher’s α), and richness (Margalef) were detected in the fungal assemblage from rock sample 5, but with a low dominance index (Simpson’s). In contrast, sample 4 generated the lowest diversity indices. Among the 92 fungal ASVs assigned, only the dominant Fungal sp. was detected in all rock samples (Fig. 4). Conversely, 35 fungal ASVs were detected exclusively in rock sample 5.

## Discussion

### Taxonomy and abundance

Our study focused on detecting the diversity and richness of endolithic fungi in the polyextreme environment of the São Pedro and São Paulo archipelago using an amplicon metagenomics approach. The eDNA recovered from the rock samples revealed high dominance of the phyla *Ascomycota* and *Basidiomycota*, as has been reported in many other amplicon metagenomics studies. We also detected the less frequently reported phyla, *Mortierellomycota* and *Chytridiomycota*, as minor components of the endolithic fungal community. Among the assigned 92 taxa, *H. werneckii*, *Cladosporium* sp., *Simplicillium* sp., *B. serpentis*, *Penicillium* sp., *P. simplicissimum*, *M. restricta*, *Ascomycota* sp., and *Verrucariaceae* sp. were dominant. These include genera commonly reported as ubiquitous (*Cladosporium* and *Penicillium*), extremophilic hypersaline (*Hortaea*), xerophilic (*Blastobotrys*), plant pathogens (*Simplicillium*) and human/animal opportunistic pathogens (*Malassezia*) [31].

*Hortaea werneckii* is a melanized yeast capable of colonizing and surviving in hypersaline environments, and is classified as a true obligate extremophile [32]. It has been detected in salterns, seawater, coral, sponges, deep-sea sediments, and the Atacama Desert [32]. Additionally, *H. werneckii* is recognized as the etiological agent of tinea nigra, which manifests as dark spots on the feet or hands of immunosuppressed individuals [33]. Recently, *H. werneckii* eDNA was detected as a dominant taxon in ornithogenic soils [31] in the São Pedro and São Paulo Archipelago, with the current study further highlighting its capability to survive and colonize different substrates and habitats in the archipelago.

The genera *Cladosporium* and *Penicillium* include a large number of species, some with worldwide distribution, which are also well known from extreme environments [34]. *Cladosporium* species are often present in xeric and hypersaline environments [35], including in the São Pedro and São Paulo Archipelago [31]. *Penicillium* species also display high capability to colonize and survive in extreme environments, including xeric and hypersaline habitats, and play important ecological roles as decomposers [36]. *Penicillium simplicissimum* has previously been reported from xeric and polluted environments and, for this reason, targeted as a potential agent in bio-lixiviation [37] and bioremediation processes [38].

The genus *Simplicillium* (*Cordycipitaceae*, *Hypocreales*) includes species occurring in diverse habitats and with various ecological roles. Members of the genus have been reported in soil, air, decaying wood, plant tissues, human nails, seawater, and rock surfaces, and display various ecological roles as symbionts, endophytes, entomopathogens, and mycoparasites [39]. The genus *Blastobotrys* includes ~ 30 species occupying different niches in soils, plants and wild animals [40]. *Blastobotrys serpentis* is a yeast isolated from the intestine of a trinket snake (*Elaphe* sp.) [41]. Kumar et al. [42] reported cases of invasive mycosis caused by *B. serpentis* in a preterm patient.

The genus *Malassezia* includes 18 species, primarily reported from human skin and guts and hospital environments, but also present in natural environments such as in deep-sea sponges [43]. Rosa et al. [44] reported eDNA of *Malassezia* taxa with high relative abundance in Antarctic soils and suggested its potential to colonize extreme environments. Gontijo et al. [45] reported the eDNA of *M. globosa* as dominant taxon in abyssal sediments obtained close to the São Pedro and São Paulo Archipelago, consistent with it being a common fungus in the archipelago.

### Fungal diversity and geological data

Fungal diversity indices varied significantly among the different rock types analyzed. Mylonitized and serpentinized peridotites exhibit similar chemical compositions, dominated by ferromagnesian silicates, with silicon, magnesium, and iron as the principal elements. This similarity reflects the hydrothermal serpentinization process, in which olivine transforms into serpentine, altering mineralogy without substantially modifying the overall rock composition. Carbonatic breccias, in contrast, are distinct due to their higher calcium content, derived from the precipitation of carbonates that cement peridotite clasts.

Despite compositional similarities, fungal diversity varied among lithotypes and within the same rock type, reflecting textural heterogeneities and the differential effects of phosphatization associated with guano deposition. In mylonitized peridotites (rock samples 1 and 2), phosphate accumulated mainly along fractured surfaces, forming crusts whose thickness depended on the degree of fracturing [1]. Rock samples with thinner crusts displayed slightly higher diversity indices, suggesting that greater fracturing promotes microhabitat formation and enhances diversity. Conversely, thicker crusts reduce permeability, which tends to restrict colonization to more specialized species.

Serpentinized peridotites exhibited both the highest and lowest diversity values. This pattern is linked to greater textural heterogeneity and deeper weathering, facilitated by the interconnected fibrous mineral networks formed during serpentinization. This microstructure enhances meteoric solution penetration, promotes saprolite formation, increases porosity, and improves water retention. More intensely serpentinized rocks, such as rock sample 5, showed higher diversity indices, whereas less affected rock samples (3 and 4) displayed lower values. Beyond the physical roles of porosity and fracturing, deeper alteration also enhances the release of chemical elements that may serve as nutrients. This highlights the combined influence of physical and chemical factors in creating favorable conditions for fungal diversity. In contrast, carbonate breccias (rock sample 6) behaved similarly to mylonitized peridotites with thick phosphate crusts. Their low porosity and more homogeneous matrix composition constrained colonization, resulting in lower diversity indices compared with other lithologies.

## Conclusions

The geochemical characteristics measured here highlight the unique nature of the sampled rocks, in particular due to the intense phosphatization process experienced. Due to is, they also may represent a challenging but viable habitat for extremophile endolithic microorganisms. Our eDNA amplicon metagenomics data indicate that the rocks of the São Pedro and São Paulo Archipelago provide microhabitats for a complex fungal diversity, including taxa reported as cosmopolitan, ubiquitous, extremophilic hypersaline and xerophilic, plant and human/animal opportunistic pathogens. As eDNA studies do not confirm the presence of viable organisms or propagules, further research, including the use of culturing approaches is now required to develop strategies to recover these fungi for physiological, biogeochemical, genetic and biotechnological studies.

## Data Availability

The datasets generated and/or analyzed during the current study are available in the NCBI repository under the codes SAMN37305790-SAMN37305802, which can be accessed at https://www.ncbi.nlm.nih.gov/.
